# Medical Robotics for Ultrasound Imaging: Current Systems and Future Trends

**DOI:** 10.1007/s43154-020-00037-y

**Published:** 2021-02-22

**Authors:** Felix von Haxthausen, Sven Böttger, Daniel Wulff, Jannis Hagenah, Verónica García-Vázquez, Svenja Ipsen

**Affiliations:** Institute for Robotics and Cognitive Systems, University of Lübeck, Ratzeburger Allee 160, 23562 Lübeck, Germany

**Keywords:** Telesonography, Collaborative robotics, Autonomous image acquisition, Autonomous therapy guidance, Intelligent systems, Virtual/augmented reality

## Abstract

**Purpose of Review:**

This review provides an overview of the most recent robotic ultrasound systems that have contemporary emerged over the past five years, highlighting their status and future directions. The systems are categorized based on their level of robot autonomy (LORA).

**Recent Findings:**

Teleoperating systems show the highest level of technical maturity. Collaborative assisting and autonomous systems are still in the research phase, with a focus on ultrasound image processing and force adaptation strategies. However, missing key factors are clinical studies and appropriate safety strategies. Future research will likely focus on artificial intelligence and virtual/augmented reality to improve image understanding and ergonomics.

**Summary:**

A review on robotic ultrasound systems is presented in which first technical specifications are outlined. Hereafter, the literature of the past five years is subdivided into teleoperation, collaborative assistance, or autonomous systems based on LORA. Finally, future trends for robotic ultrasound systems are reviewed with a focus on artificial intelligence and virtual/augmented reality.

## Introduction

Ultrasound has become an indispensable medical imaging modality for both diagnostics and interventions. As a radiation-free, portable, widely available, and real-time capable imaging technique, this imaging modality has significant advantages compared to other techniques such as computed tomography (CT) or magnetic resonance imaging (MRI). Additionally, real-time volumetric ultrasound (four-dimensional, 4D) has recently gained attention as new matrix array probes provide sufficiently high frame rates for many medical applications. However, ultrasound is a strongly user-dependent modality that requires highly skilled and experienced sonographers for proper examinations. Apart from identifying the correct field of view, thus being continuously focused on the ultrasound station screen, and holding the probe manually with an appropriate pressure, the examiner must also adjust several imaging settings on the ultrasound station. This un-ergonomic examination process may also lead to work-related musculoskeletal disorders [1, 2]. Further, manual guidance of the probe makes reproducible image acquisition almost impossible. While spatially and temporally separated image acquisition and diagnostics are common practice for MRI and CT, sonographers must perform both at the same time, making the examination mentally more demanding.

Robotic ultrasound is the fusion of a robotic system and an ultrasound station with its probe attached to the robot end-effector. This combination might overcome ultrasound disadvantages by means of either a teleoperated, a collaborative assisting, or even an autonomous system. A range of commercial and research systems have been developed over the past two decades for different medical fields, and many were summarized in previous reviews [3, 4]. Nevertheless, this review focuses on the most recent systems with the emphasis on findings published in the last five years, highlighting the current status and future directions of robotic ultrasound. We use the level of robot autonomy (LORA) [5] to organize the sections of this review into either teleoperated, collaborative assisting, or autonomous systems. In addition, each described system was objectively classified to a LORA between level one and nine after defining the task to be performed autonomously by the robotic ultrasound systems as: The ultrasound acquisition of a specific anatomical region of interest (ROI) including the initial placement of the ultrasound probe. The LORA values correspond to the following terms (further information on the levels in Fig. 6, Appendix 1):

*Teleoperation:*

1. Teleoperation
2. Assisted Teleoperation

*Collaborative assistance:*

3. Batch Processing
4. Decision support

*Autonomous systems:*

5. Shared control with human initiative
6. Shared control with robot initiative
7. Executive control
8. Supervisory control
9. Full autonomy

This review starts by presenting the technical specifications and requirements for these systems with a focus on ultrasound imaging and safety considerations of the robot. The reviewed systems are then categorized into teleoperation, collaborative assistance, and autonomous systems. Finally, an outlook for future directions of robotic ultrasound systems combined with artificial intelligence (AI) or virtual/augmented reality (VR/AR) is provided, as these technologies have gained increased attention in the past years. AI-based applications can achieve exceptional performance in medical image understanding which could be crucial for increasing autonomy of robotic ultrasound systems. VR/AR, on the other hand, may facilitate an enhancement of the physician’s perception with subsurface targets and critical structures while also potentially improving 3D understanding.

## Technical Specifications

### Ultrasound Imaging

Using a robot to perform ultrasound imaging poses task-specific challenges for the imaging system. If the task of the robotic ultrasound system requires visual servoing (the process of controlling robot motion based on image information [6, 7]), online data access is mandatory. In case of two-dimensional (2D) ultrasound images, data can usually be accessed by grabbing the frames at the display output of the ultrasound system. In contrast, volumetric data offer the distinct advantage of covering entire anatomical structures, and their motion paths can then be used for automated robotic control. However, three-dimensional (3D) data are more complex and therefore require a dedicated interface for streaming. Robotic ultrasound imaging might also require remote or even automatic control of the imaging parameters which are usually adjusted manually on the ultrasound system. Remote control, just like direct data access, is typically not enabled by commercial diagnostic systems and thus requires development of open platforms or close collaborations with manufacturers for integration.

### Force Sensitivity and Safety Considerations

Medical robotic ultrasound sets special safety requirements beyond the established industry standards of human-robot collaboration where direct contact between the robot and humans is typically to be avoided. Patients, who are purposely touched by the moving robot tool, are in an unprotected position with no quick escape possibility from the dangerous area and are possibly physically weakened. The potential dangers to patient and personnel during robot operation are clamping, squeezing, impact, and pressing in various ways. These dangers can be detected by extensive technical precautions on the robot system and should be prevented or stopped early at the onset of a potential injury.

Safety technologies usually contain either external force/torque sensors mounted on the end-effector or, in the case of lightweight robots, integrated torque sensors in all joints, realizing proprioceptive sensing. While the former does not allow to perform collision checks of the arm links, the latter calculates the contact force at the end-effector and possible collision forces at the individual arm links by means of a dynamic model with the joint torque measurements. Moreover, this technique enables the modeling of impedance/admittance-controlled motion modes that mimic the behavior of a multidimensional spring-damper system, enabling a safer human-robot interaction. Lightweight robots also have the advantage of taking up lower kinetic energy and thus potentially reducing the risk of injury. Camera surveillance and the integration of external proximity sensors can also reduce the risks but are more expensive to implement and to maintain and can also be adversely affected by interruptions of the direct line-of-sight. In addition, research is also being conducted on mechanical safety concepts that intrinsically protect against hazards [8, 9].

Dynamic concepts of injury prevention consist of adapted velocity profiles depending on the distance to the patient and the blocking of safe areas against robot movement. Additionally, the anticipation and treatment of collisions in the application context through a structured process in real time could be used to prevent adverse events [10]. The fast and often short-term nature of collisions requires maximal detection and data processing speed. The main problem of collision detection is signal monitoring with high sensitivity while also avoiding false alarms.

Safety aspects are often not the primary focus in many research projects. Nevertheless, meeting these safety requirements should be considered already during the conception and development phases of a project to ensure safe operation and facilitate a subsequent product certification.

## Teleoperation

The operator dependency of ultrasound imaging means that receiving a reliable diagnosis generally depends on the availability of an expert sonographer. Considering the shortage of trained experts especially in remote regions, access to ultrasound imaging can be very limited, increasing travel and waiting times with potential negative effects on patient outcomes. Another problem is the physical strain of manually handling the probe [1, 2]. Remote control of the ultrasound probe using robotic technology (LORA one and two) holds the potential to solve these problems. In this section, the most recent systems are categorized into custom design and commercially available robotic hardware and summarized in Table 1.

**Table 1:** Overview of teleoperated and collaborative robotic ultrasound systems and their respective components, published between 2015 and 2020

Topic	System/institution	Medical field(s)	Robot		Key system components			In vivo/phantom study	LORA	Year	Ref.
			Type	Force measurement	Ultrasound probe	Camera system	User interface				
Teleoperation	*MGIUS-R3*, Shenzhen, China	Not specified	Serial; 6 DOF; custom design	External F sensor	Not specified	Not specified	Dummy probe (6 DOF, robot control), keyboard (US control)	Patient volunteer (1)	1	2020	[13]
	*MELODY*, Naveil, France	Cardiac, abdominal, vascular, pelvic, urinary, prenatal	Serial; 3 active DOF rotation, 3 passive DOF translation (manual); custom design	External F sensor; 20 N limit	Convex probe; *C5-2* [BK Medical]	RGB camera (webcam conferencing)	Haptic device (6 DOF, custom, robot control), tablet/keyboard (US control)	Patient volunteers (> 300)	2	2016; 2017; 2018	[14–16, 17•]
	*ReMeDi*, Lublin, Poland	Cardiac, abdominal	Serial; 7 active DOF; custom design to reproduce human examiner	External FT sensor (6 DOF); max. force 40 N	Not specified	RGB camera (webcam conferencing)	Haptic device (6 DOF, custom, robot control)	Patient volunteers (> 10)	2	2016; 2017; 2019; 2020	[18–20, 21••]
	TOURS (Sonoscanner-CNES), Tours, France	Abdominal, vascular, pelvic, prenatal	Parallel; 3 active DOF rotation, translational placement manual; custom design	None	3D convex probe and linear probes; [Vermon]	RGB camera (webcam conferencing)	Dummy probe (robot control), keyboard (US control)	Patient volunteers (> 100)	1	2016; 2018	[22, 23•]
	*ROBO Health Institute,* Shenzhen, China	Not specified	Serial; 6 DOF (× 3 prismatic, × 3 revolute); custom design	External F sensor (6 DOF)	Convex probe; [SonoStar]	RGB camera (webcam conferencing)	Keyboard (robot translation), dummy probe (robot rotation)	Healthy volunteer (1)	2	2017	[24]
	*CNMC,* Washington, USA	Not specified	Parallel; 6 DOF (fine positioning); custom design	External FT sensor (6 DOF)	Convex probe; *4C2* [Terason]	RGB camera (webcam conferencing)	Haptic device (6 DOF, [Phantom, Omni], robot control)	Phantom	2	2015	[25]
	*ROBIN,* Oslo, Norway	Not specified	Serial; 6 DOF; *UR5* [Universal Robots]	Internal T sensors; external F sensor (6 DOF)	Linear probe; [GE]	Not specified	Haptic device (6 DOF, [Phantom, Omni], robot control)	Phantom	2	2016	[26]
	*BME-SJTU,* Shanghai, China	Not specified	Serial; 6 DOF; *UR5* [Universal Robots]	Internal T sensors	Convex array probe; *CLA3.5* [Vermon & prototype US]	Not specified	Haptic device (6 DOF, [Touch, 3D System Corp.], robot control)	Phantom	2	2020	[27]
	*ISR,* Coimbra, Portugal	Not specified	Serial; 7 DOF; *WAM* [Barrett]	External F sensor (6 DOF)	Convex probe; *Titan C15* [SonoSite]	Monochrome-D camera; *CamBoard nano* [pmd[vision]]	Haptic device (6 DOF, [Phantom, Omni], robot control)	Healthy volunteer (1)	2	2015; 2018	[28, 29]
	*OPTIMAL,* Xi’an, China	Vascular	Serial; 6 DOF; *C4* [Epson]	None	Convex and linear probe; *C7-3/50* and *L9-4/38* [Ultrasonix]	4 RGB cameras (webcam conferencing)	Joystick (controller)	Healthy volunteer (1)	1	2019	[30]
Collaborative assistance	*CRCHUM,* Montréal, Canada	Iliac-popliteal artery	Serial; 6 DOF; CRS *F3* [Thermo CRS]	None	Linear probe; *FLA-10* [GE] and *L12-5* [Philips]	None	Display of volume rendering	Patient volunteers (2)	4	2014	[32]
	*CAMP,* Munich, Germany	Orthopedic	Serial; 7 DOF; *LBR iiwa* [KUKA]	Internal T sensors	Convex probe; *C5-2/60* [Ultrasonix]	None	Not specified	Healthy volunteer (1)	4	2020	[33]
		Soft tissue	Serial; 7 DOF; *LBR iiwa* [KUKA]	Internal T sensors	Linear probe; [Ultrasonix]	None	Not specified	Healthy volunteers (30)	4	2018	[34•]
		Spine	Serial; 7 DOF; *LBR iiwa* [KUKA]	Internal T sensors	Convex probe; *C5-2/60* [Ultrasonix]	None	Display of volume rendering	Patient volunteers (2)	3	2018	[39•]
	*Johns Hopkins,* Baltimore, USA	Not specified	Serial; 6 DOF; *UR5* [Universal Robot]	External FT sensor (6 DOF) [Robotiq]	Convex probe; *C5-2* [Ultrasonix]	None	Hand guidance with virtual walls	Phantom	4	2016	[35]
	*PLA,* Beijing, China	Liver	Serial; 6 DOF; *UR5* [Universal Robot]	None	Convex probe; [Minddray]	None	Controller and display unit	Animal tissue	4	2018	[36]
	*Rutgers,* New Brunswick, USA	Forearm vessels	Serial; 3 DOF; custom design	Not specified	Linear probe; [Telemed]	NIR camera; custom design	Not specified	Healthy volunteers (9)	4	2016	[37•]
	*IRISA,* Rennes, France and TUM, Munich, Germany	Generic needle guidance	× 2 serial; 6 DOF; *Viper s650* [Adept]	Not specified	3D convex probe; *4DC7-3/40* [Ultrasonix]	None	Display of highlighted needle position	Phantom	4	2015	[38]
	*IPCB,* Castelo Branco, Portugal	Femoral orthopedic	Serial; 7 DOF; *LBR* [KUKA]	None	Linear probe; [Aloka]	Tracking camera; *Polaris Spectra* [NDI]	Not specified	Phantom	4	2015	[40]

*F* force, *FT* force-torque, *max* maximum, *Ref* references, *T* torque, *US* ultrasound

### Custom Design Robots

**Fig. 1:**
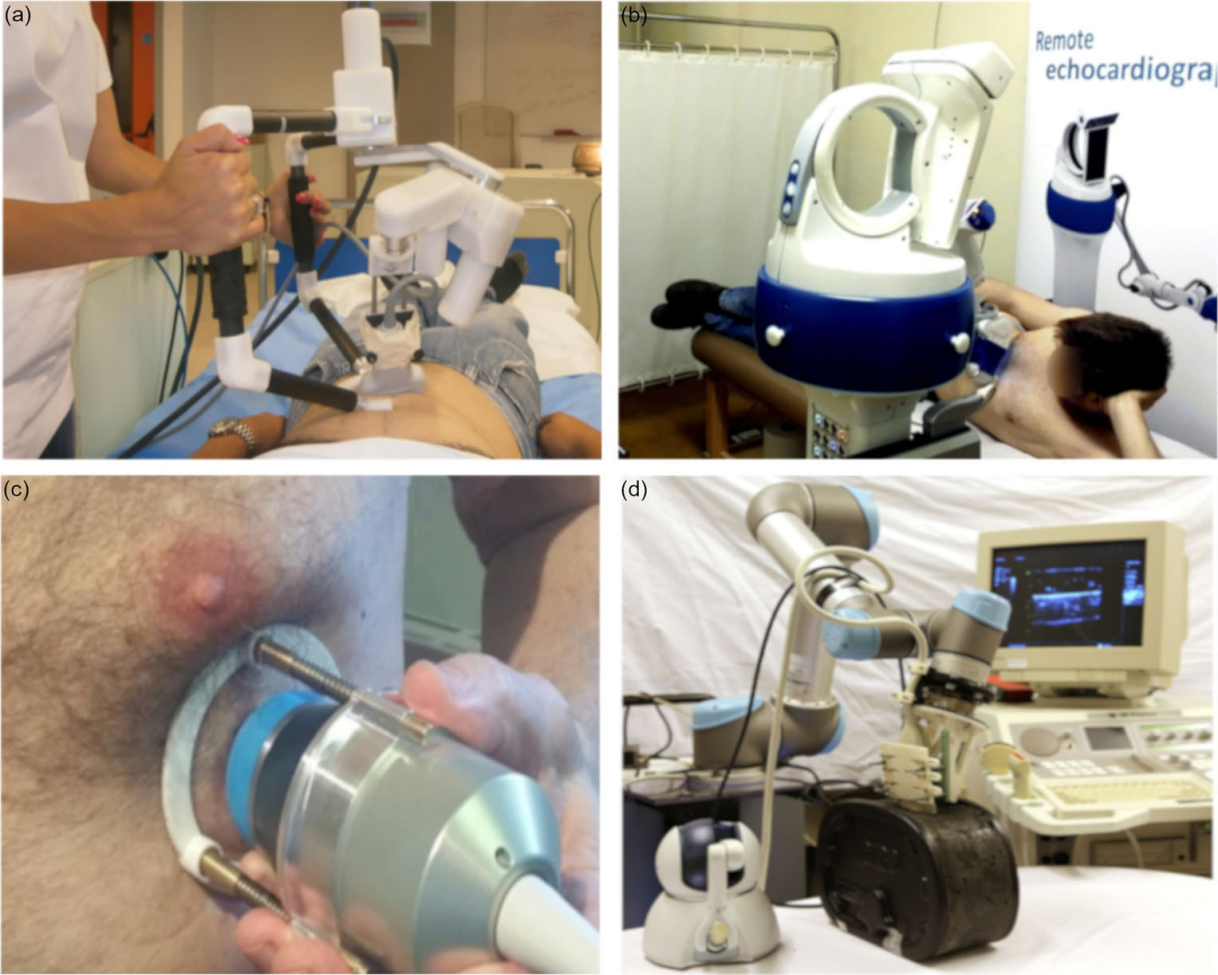
Overview of different teleoperated robotic ultrasound systems. **a** *MELODY* system used in an abdominal exam (picture courtesy S. Avgousti, Cyprus University of Technology). **b** *ReMeDi* system used in a cardiac exam (figure by M. Giuliani et al. [21••] under CC-BY license). **c** *TOURS* system as utilized for remote exams on the International Space Station (reprinted from [23•], copyright [2018], with permission from Elsevier). **d** Teleoperated ultrasound platform with haptic device while acquiring an imaging phantom (figure by K. Mathiassen et al. [26] under CC-BY license)

The only commercially available teleoperated ultrasound solutions to date are the *MGIUS-R3* (MGI Tech Co.) system [11] and the MELODY (AdEchoTech) system [12]. The former system consists of a six degrees of freedom (DOF) robotic arm including a force sensor and the ultrasound probe. A dummy probe (simple model made from plastic) at the physician site allows controlling the actual probe at the remote site. A single study was conducted to assess the feasibility of examining a patient with COVID-19, highlighting its advantage regarding the eliminated infection risk for the physician [13]. MELODY consists of a specialized robotic probe holder at the patient site (Fig. 1a) with three passive DOF for positioning, three active DOF for rotating the probe, and a force sensor. Coarse translational positioning of the robot is handled by a human assistant, while fine adjustments of probe orientation are remotely controlled by the expert sonographer via a haptic device with force feedback. MELODY has already been used for cardiac [14], abdominal [15, 16], obstetric [15, 17•], pelvic, and vascular telesonography [15] in over 300 patients.

The novel ReMeDi (Remote Medical Diagnostician) system is based on a detailed analysis of user requirements with a focus on safety, dexterity, and accurate tactile feedback [18, 19]. The kinematically redundant robotic arm (Fig. 1b) features seven active DOF and an additional force-torque sensor and was specially designed to reproduce all necessary movements of a human examiner [20]. In contrast to MELODY, ReMeDi does not rely on a human assistant. This system has successfully been tested in 14 patients for remote cardiac exams [21••].

The *TOURS* (Tele-Operated UltRasound System) features a compact robotic probe manipulator (Fig. 1c) with three active DOF for remote control of probe orientation via a dummy probe without haptic feedback [22]. Translation is handled manually by an assistant at the patient site. *TOURS* has been tested over long distances for abdominal, pelvic, vascular, and obstetric exams in over 100 patients [22]. The system has also been successfully employed for remote ultrasound scans on the International Space Station [23•].

In [24], a specially designed robot with six DOF and a force sensor was controlled using a dummy probe for probe rotations and a conventional keyboard for translational motion. Feasibility was demonstrated in a healthy volunteer. A compact parallel telerobotic system with six DOF for fine positioning of the probe and haptic feedback for remote control was presented in [25] but not tested in vivo yet.

### Commercial Robots

In [26], the six DOF *UR5* robot (Universal Robots) was used to develop a general, low-cost robotic ultrasound platform. The integrated torque measurements were enhanced with an external force sensor, and a haptic device was used for remote control (Fig. 1d). The system meets the technical requirements for teleoperated ultrasound, but has not been evaluated in vivo [26]. A similar study using the *UR5* robot investigated filtering haptic commands and reducing velocity to improve safety [27].

A new control approach was presented in [28, 29] using a lightweight anthropomorphic robot (*WAM*, Barrett Technology) with seven DOF and remote control with a haptic device. To achieve smooth transitions between free movement and patient contact, an external force sensor and a 3D time-of-flight camera were integrated. The architecture was validated in a pelvic exam of a healthy volunteer with the examiner located in the same room.

In [30], a ProSix C4 robot (Epson) without force sensors was proposed for acquiring ultrasound images for 3D volume reconstruction using remote control of the probe via joystick. Safety and surveillance relied on visual inspection by the operator via camera. The authors tested their setup for a vascular scan on a healthy volunteer.

### Summary

The past five years have proven feasibility of performing remote ultrasound exams of various anatomical regions at varying distances. Patients and examiners generally accept this new technology [21••], which could improve access to care, for example, by reducing waiting times for a consultation in remote locations which lack experienced sonographers [31].

## Collaborative Assistance

Research in the field of collaborative robotic ultrasound assistance typically aims to enable physicians to perform standard ultrasound imaging procedures faster, more precise, and more reproducible. On the other hand, collaborative therapy guided interventions may be performed with reduced assistant personnel or even alone. In this review, collaborative assisting robotic ultrasound systems comprise systems that have a LORA of three and four and thus can perform a certain action and partially even suggest a task plan. This section introduces applications and functionality of such systems, while Table 1 shows an overview of the most important recent systems.

### Collaborative Image Acquisition

**Fig. 2:**
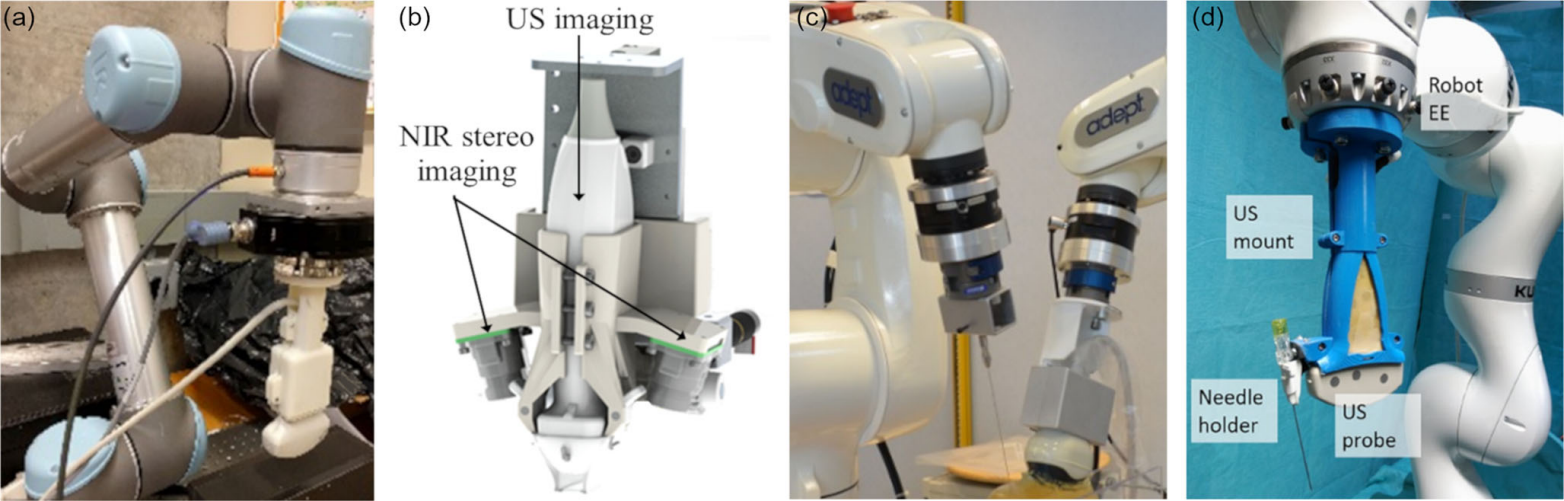
Overview of system components for collaborative assisting robotic ultrasound systems. **a** The *STRATUS* system including a *UR5* robot and an ultrasound probe interconnected by a six DOF force-torque sensor (copyright © [2016] IEEE. Reprinted with permission from [35]). **b** Near infrared imaging sensors combined with an ultrasound probe for bimodal vessel imaging in the forearm to guide venipuncture (reproduced from [37•] with permission from Springer Nature). **c** Setup for a flexible needle steering system of two *Viper s650* robots (Adept) with needle holder and ultrasound probe (copyright © [2015] IEEE. Reprinted with permission from [38]). **d** *LBR iiwa* robot with ultrasound probe on custom mount with needle holder used for facet joint insertion (reproduced from [39•] with permission from Springer Nature)

The reconstruction of the iliac artery has been performed by Janvier et al. [32] using their system of a six DOF *CRS F3* robot (Thermo CRS) with an attached linear probe, whereby the scan path over the ROI was manually taught and the vessel surface structure was reconstructed from multiple automatically replayed robotic cross-sectional ultrasound scans. The authors compared the ultrasound volume reconstruction from the system to computed tomography angiographies of a phantom and in vivo. Ultrasound image quality was optimized by Jiang et al. [33] by adjusting the in-plane and out-of-plane orientation of the ultrasound probe. Therefore, an initial confidence map of the ultrasound image was analyzed, and a subsequent fan motion was then automatically performed with a force-sensitive *LBR iiwa* robot (KUKA). A method for the correction of contact pressure-induced soft-tissue deformation in 3D ultrasound images was developed by Virga et al. [34•]. The image-based process utilizes displacement fields in a graph-based approach which in turn is based solely on the ultrasound images and the applied force measured by the robot. Zhang et al. applied the concept of synthetic tracked aperture ultrasound (STRATUS) in [35] to extend the effective aperture size by means of robotic movements (Fig. 2a). During the process, the system accurately tracks the orientation and translation of the probe and improves image quality especially in deeper regions. Here, sub-apertures captured from each ultrasound pose were synthesized to construct a high-resolution image. Thereby, the probe has been moved by an operator, while a virtual wall for constraining the motion to the desired image plane is mimicked by the force feedback control of an external force-torque sensor.

### Collaborative Therapy Guidance

A system for needle insertion and needle guidance during the ablation of liver tumors was developed by Li et al. [36], utilizing a robotic ultrasound system with real-time imaging and respiratory motion compensation. Chen et al. [37•] reported the use of automatic image segmentation, reconstruction, and motion tracking algorithms for the ultrasound probe, which is mechanically connected to near infrared sensors and forms a portable device (Fig. 2b). The system shall perform robotic venipuncture but has so far only been validated for manually guided procedures in forearm vessels. Robotized insertion and steering of a flexible needle in a phantom under 3D ultrasound guidance with one robot for needle steering and a second robot for ultrasound imaging (Fig. 2c) were performed by Chatelain et al. [38]. In 2018, Esteban et al. reported the first clinical trial of a robotized spine facet joint insertion system in [39•], performing a force-compliant sweep over the spine region with automatic volume reconstruction to facilitate intrainterventional insertion planning and subsequent precise needle prepositioning over the target. The system consists of a calibrated probe holder with a needle guide mounted on an *LBR iiwa* robot (Fig. 2d). A navigation assistant for markerless automatic motion compensation in a custom femur drilling *LBR* robot (KUKA) was developed by Torres et al. [40] and evaluated on a bone phantom. The dynamic bone position and orientation were registered intrainterventionally with the image of a manually operated optically tracked ultrasound probe, and a preinterventional CT scan in which the target was defined.

### Summary

Research in recent years was performed in the areas of optimization for probe alignment, 3D tissue reconstruction, anatomical target recognition, vessel segmentation, and tracking. Intensive work has been done to replace external force sensors, adapt the force control for lightweight robots, improve motion compensation and trajectory planning, accelerate real-time imaging, and refine calibration. Resulting systems provide more comfort with less fatigue for the operator and improved image quality compared to conventional ultrasound.

## Autonomous Systems

Autonomous systems in the field of robotic ultrasound may be considered to be systems facilitating independent task plan generation and consequent control and movement of the robot to acquire ultrasound for diagnostics or interventional tasks. First, autonomous image acquisition systems and, afterwards, systems for autonomous therapy guidance with respect to the medical fields of minimally invasive procedures, high-intensity focused ultrasound (HIFU), and radiation therapy are reviewed in this section. The systems described in this section may have a LORA between five and nine. However, the highest LORA obtained in this review is seven. The systems are presented in Table 2.

**Table 2:** Overview of autonomous robotic ultrasound systems and their respective components, published between 2015 and 2020

Topic	Subtopic	Institution	Medical field(s)	Robot		Key system components		Data source for autonomous control	In vivo/phantom study	LORA	Year	Ref.
				Type	Force measurement	Ultrasound probe	Camera system					
Autonomous image acquisition	3D image reconstruction	CRCHUM, Montréal, Canada	Vascular	Serial; 6 DOF; *F3* [CRS]	External FT sensor (6 DOF)	Linear probe; *L14-7* [Ultrasonix]	None	US image; force	Phantom; healthy volunteer (1)	6	2016	[41]
		OPTIMAL, Xi’an, China	Not specified	Serial; 6 DOF; *C4* [EPSON]	Two external F sensors	Linear probe; *L9-4/38* [Ultrasonix]	RGB-D camera; *Kinect* [Microsoft]	Force; camera	Phantom	7	2019	[42]
	Trajectory planning and probe positioning	CAMPAR, Munich, Germany	Vascular	Serial; 7 DOF; *LBR iiwa* [KUKA]	Internal T sensors	3D convex probe; *4DC7-3/40* [Ultrasonix] single plane mode	RGB-D camera; *Kinect* [Microsoft]	MRI image; US image; force; camera	Phantom; healthy volunteers (2)	7	2017	[43]
			Vascular	Serial; 7 DOF; *LBR iiwa* [KUKA]	Internal T sensors	3D convex probe; *4DC7-3/40* [Ultrasonix]; single plane mode	RGB-D camera; *Kinect* [Microsoft]	MRI image; US image; force; camera	Healthy volunteers (5)	7	2016	[44•]
			Not specified	Serial; 7 DOF; *LBR iiwa* [KUKA]	Internal T sensors	Convex probe; *C5-2* [Ultrasonix]	RGB-D camera; *Kinect* [Microsoft]	MRI image; force; camera	Phantom; animal tissue	7	2016	[45]
			Thyroid	Serial; 7 DOF; *LBR iiwa* [KUKA]	Internal T sensors	Linear probe; *L14-5* [Ultrasonix]	RGB-D camera; *RealSense SR300* [Intel]	Force; camera	Healthy volunteers (4)	7	2017	[46]
		Institute for Robotics and Cognitive Systems, Lübeck, Germany	Vascular	Serial; 7 DOF; *LBR iiwa* [KUKA]	Internal T sensors	Linear probe; *L12-3* [Philips]	None	US image; force	Phantom	6	2020	[47•]
		IRISA, Rennes, France	Not specified	Serial; 6 DOF; *Viper 850* [Adept]	External F sensor	3D convex probe; *4DC7-3/40* [Ultrasonix]	None	US image; force	Phantom	6	2016	[48]
	Image quality improvement	IRISA, Rennes, France and CAMPAR, Munich, Germany	Not specified	Serial; 6 DOF; *Viper s650* [Adept]	External FT sensor	3D convex probe; *4DC7-3/40* [Ultrasonix]	None	US image; force	Phantom	5	2016	[49]
			Not specified	Serial; 7 DOF; *LBR iiwa* [KUKA]	Internal T sensors	3D convex probe; *4DC7-3/40* [Ultrasonix]	None	US image; force	Phantom; healthy volunteer (1)	6	2017	[50•]
Autonomous therapy guidance	Tool tracking	CAMP, Munich, Germany	Vascular	Serial; 7 DOF; *LBR iiwa* [KUKA]	Internal T sensors	Convex probe; *C5-2* [Ultrasonix]	None	US image; force	Phantom	6	2019	[51•]
	Target tracking	CAMP, Baltimore, USA and CMBR, Pontedera, Italy	Needle placement	× 2 serial; 7 DOF; *LBR iiwa* [KUKA]	Not specified	Convex probe; *C5-2* [Ultrasonix]	RGB-D camera; *RealSense F200* [Intel]	US image; camera	Phantom	7	2016	[52]
		UNISTRA, Strasbourg, France	HIFU	Serial; 6 DOF; [Sinters]	None	2D probe; not specified	NA	US image	Phantom	6	2015	[54]
		NCU, Taoyuan, Taiwan	HIFU	Serial; 4 DOF; *YK400XG* [YAMAHA]	None	2D probe; not specified	Tracking camera; *Polaris Spectra* [Northern Digital]	US image, Camera	Phantom	5	2017	[55]

*F* force, *FT* force-torque, *Ref* references, *T* torque, *US* ultrasound

### Autonomous Image Acquisition

Autonomous image acquisition systems are categorized into the following three main objectives: (1) using robotic ultrasound systems to create a volumetric image by combining several images and spatial information, (2) autonomous trajectory planning and probe positioning, and (3) optimizing image quality by probe position adjustment.

#### 3D Image Reconstruction

**Fig. 3:**
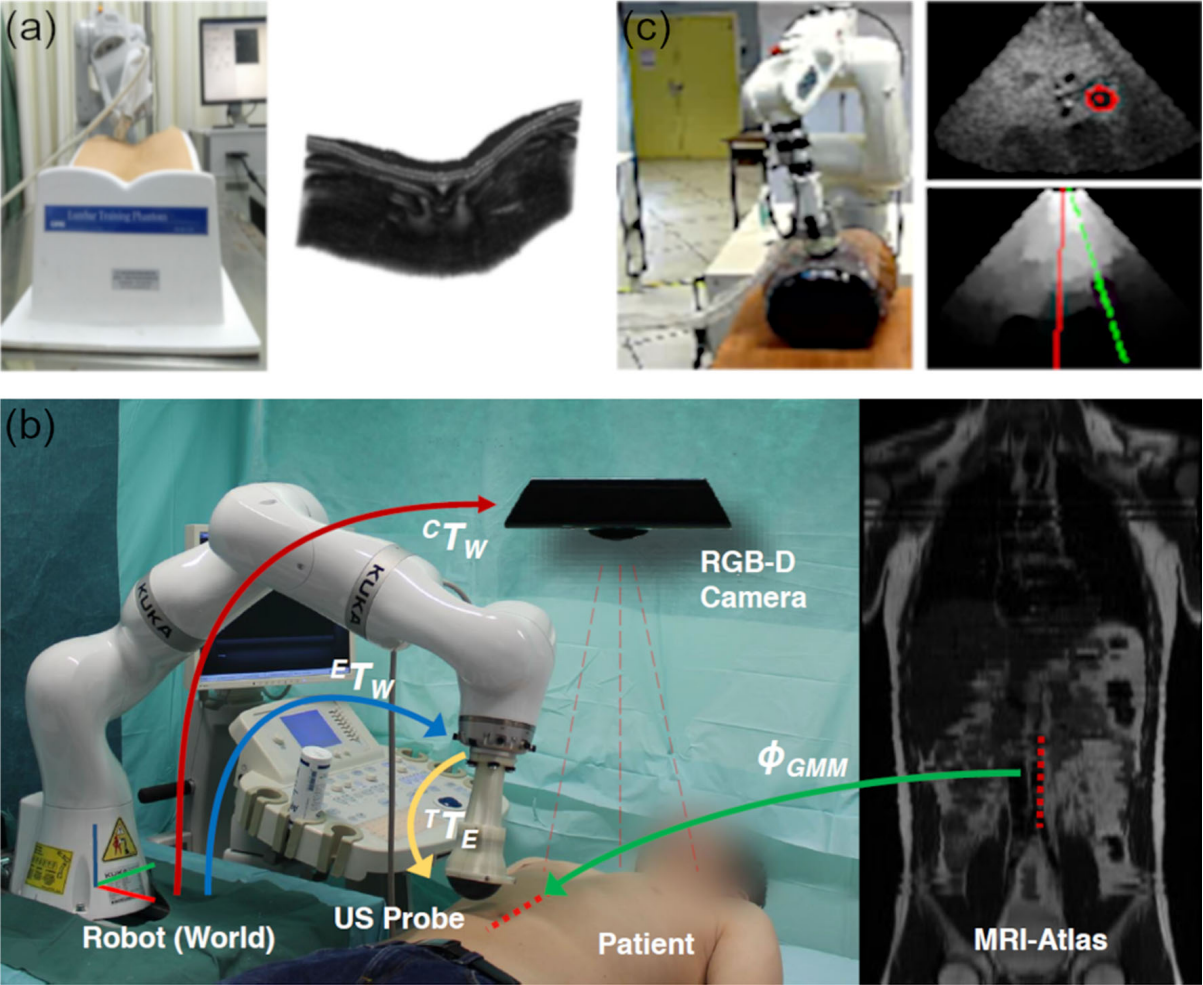
Overview of different robotic ultrasound systems for autonomous image acquisition. **a** A robotic ultrasound system autonomously scanning along a lumbar phantom (left) and the reconstructed ultrasound volume from 2D images (right) (copyright © [2019] IEEE. Reprinted with permission from [42]). **b** System setup including transformations (arrows) between robot, camera, ultrasound probe, and patient (left). MRI atlas displaying the generic trajectory (dotted red line) to image the aorta (right) (copyright © [2016] IEEE. Reprinted with permission from [44•]). **c** Robotic ultrasound system and phantom (left) with the target (red) in the ultrasound image (top right). A confidence map is calculated, and the current and desired configuration (red and green line, respectively) are calculated (bottom right) (copyright © [2016] IEEE. Reprinted with permission from [49])

A robotic ultrasound system to reconstruct peripheral arteries within the leg using 2D ultrasound images and an automatic vessel tracking algorithm was developed in [41]. The physician initially places the probe on the leg such that a cross-section of the vessel is visible. Thereafter, the vessel center is detected, and the robotic arm moves autonomously such that the vessel center is in the horizontal center of the image. A force-torque sensor is placed between probe holder and end-effector that allows keeping a constant pressure during the scan. The 3D reconstruction was performed online during the acquisition. Huang et al. [42] presented a more autonomous system that encompasses a depth camera in order to identify the patient and independently plan the scan path of the ultrasound robot. After spatial calibration, the system could autonomously identify the skin within the image and scan along the coronal plane using a normal vector-based approach for probe positioning (Fig. 3a). Two force sensors placed at the bottom of the probe ensured proper acoustic coupling during image acquisition.

#### Trajectory Planning and Probe Positioning

Hennersperger et al. [43] developed a robotic ultrasound system using an *LBR iiwa* robot that can autonomously conduct trajectories based on selected start and end points selected by a physician in preinterventional images such as MRI or CT. Given the start and end points within the MRI data, the trajectory was calculated by computing the closest surface point and combining it with the corresponding surface normal direction. Drawbacks of this method are the need for patients to hold their breath and the necessity of preinterventional image acquisition prior to selecting start and end points. The same research group overcame this drawback and used the system for quantitative assessment of the diameter of the abdominal aorta [44•]. Based on an MRI atlas and the registration to the current patient, the robot follows a generic trajectory to cover the abdominal aorta (Fig. 3b). An online force adaptation approach allowed measuring the aortic diameter even while the patient was breathing during acquisition. The system setup proposed by Graumann et al. [45] was similar but with the main objective to autonomously compute a trajectory in order to cover a volume of interest within previously obtained images such as CT, MRI, or even ultrasound. The robotic ultrasound system could cover the volume by single or multiple parallel scan trajectories. Kojcev et al. [46] evaluated the system regarding the reproducibility of measurements performed by the system producing ultrasound volumes compared to an expert-operated 2D ultrasound acquisition.

Von Haxthausen et al. [47•] developed a system that, after a manual initial placement of the probe, can control the robot in order to follow peripheral arteries, whereas the vessel detection is realized using convolutional neural networks (CNNs).

A system that provides an automatic probe position adjustment with respect to an object of interest was proposed in [48]. Their approach is based on visual servoing using image features (image moments). The authors used a 3D ultrasound probe and extracted features from the three orthogonal planes to servo in- and out-of-plane motions.

#### Image Quality Improvement

Since ultrasound imaging suffers from high user dependency, there is a strong interest in autonomously improving the image quality by means of probe positioning of the robot. Chatelain et al. dedicated several publications to this topic. The authors proposed a system that can automatically adjust the in-plane rotation for image quality improvement while using a tracking algorithm for a specific anatomical target [49]. The main objective was to keep the object horizontally centered within the ultrasound image while scanning the best acoustic window for the object (Fig. 3c). However, out-of-plane control is not considered. Their following work [50•] utilized the same approaches but for an ultrasound volume instead of a 2D image that in turn could provide tracking and image quality improvement for all six DOF.

#### Summary

Several systems and approaches have been proposed to provide autonomous image acquisition with respect to 3D image reconstruction, trajectory planning, probe positioning, and image quality improvement. A key component for initial autonomous probe placement is a depth camera to capture relative positions of robot and patient. Mostly, preinterventional images such as CT or MRI were used to calculate the trajectory needed to image the desired volume of interest. To improve image quality during acquisition, the systems rely on ultrasound image processing and force information. Even though some studies provide in vivo results, safety aspects with respect to the workflow are rarely considered within the reviewed articles.

### Autonomous Therapy Guidance

This subsection presents systems that eliminate the need of human intervention for imaging during therapy. Using an autonomous system has the benefit that the physician can concentrate on the interventional task while a robot performs ultrasound imaging. To realize this, ultrasound images need to be interpreted automatically to be able to continuously track and visualize the ROI for guidance.

#### Minimally Invasive Procedures/Needle Guidance

**Fig. 4:**
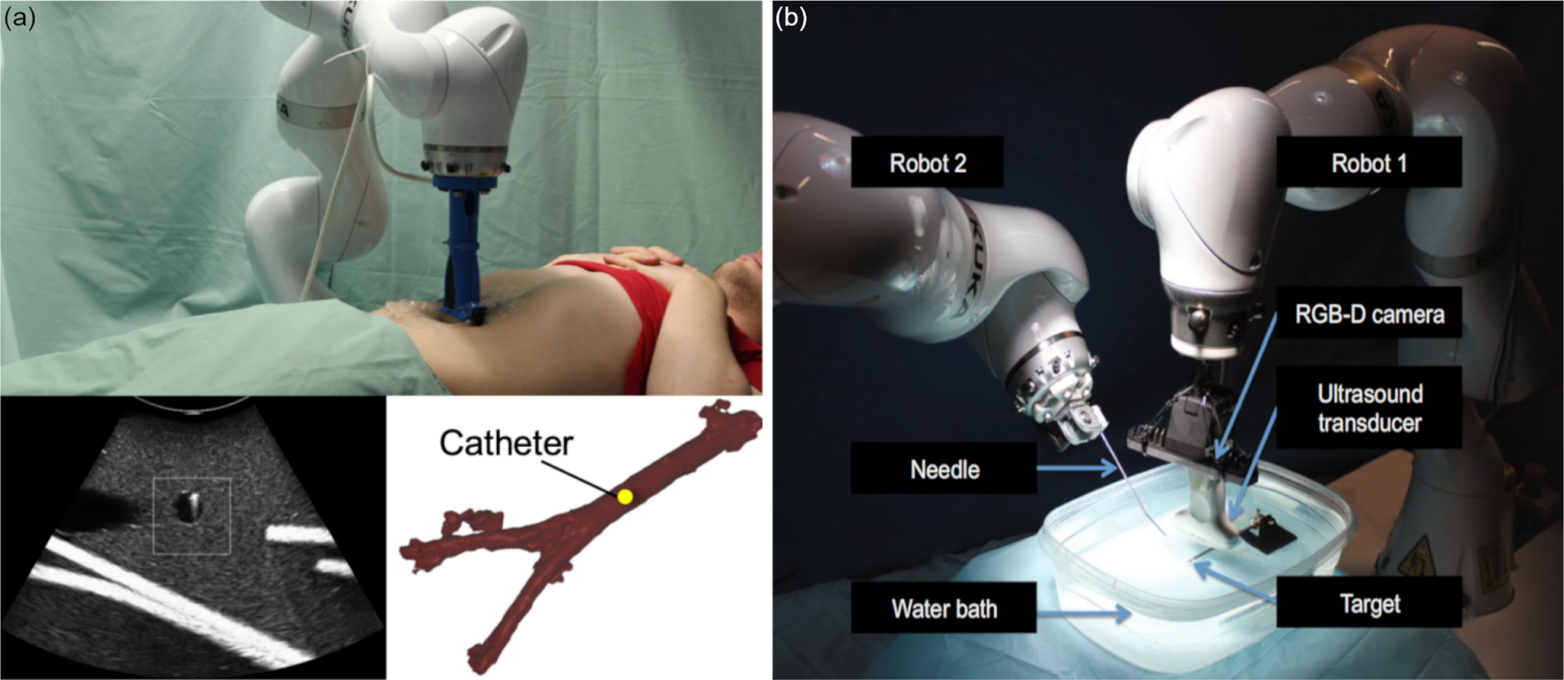
Examples of autonomous therapy guidance systems. **a** Autonomous robotized catheter tracking for EVAR with an *LBR iiwa* robot. Robot ultrasound setup (top), ultrasound image (bottom left), and 3D vessel model (bottom right) (copyright © [2019] IEEE. Reprinted with permission from [51•]). **b** Dual-robot system with two *LBR iiwa* robots performing both target tracking and needle insertion in a water bath phantom (reproduced from [52] with permission from Springer Nature)

In [51•], the authors proposed an autonomous catheter tracking system for endovascular aneurysm repair (EVAR). As illustrated in Fig. 4a, an *LBR iiwa* robot with a 2D ultrasound probe is used to acquire ultrasound images. In a preinterventional CT, the vessel structure of interest is segmented and subsequently registered to the intrainterventional ultrasound images. During the intervention, a catheter is inserted into the abdominal aorta by a physician, and the endovascular tool is guided to the ROI. The robot follows the catheter using a tracking algorithm and force control law so that the catheter tip is continuously visible in the ultrasound images. For needle placement tasks such as biopsies, Kojcev et al. [52] proposed an autonomous dual-robot system (Fig. 4b). The system can perform both ultrasound imaging and needle insertion. In this phantom study, two *LBR iiwa* robots are used, one holding the needle and the other one holding the ultrasound probe. Preinterventional planning data is registered to the robot coordinate system in the initialization phase using image registration. The physician selects the ROI on the patients’ surface images acquired by RGB-D (depth) cameras mounted on the robots. The robots move the ultrasound probe and the needle to the ROI and start target tracking based on a predefined target and also needle tracking to perform needle insertion as planned. A dual-robot system provides higher flexibility than a one-robot system as used in [39•, 53], but its setup is more complicated to implement.

#### High-Intensity Focused Ultrasound

Another application field is tumor treatment with HIFU. In [54], one 2D ultrasound probe and the HIFU transducer are mounted to a six DOF robotic arm. The HIFU focus is adapted by using speckle tracking to determine the difference between target and HIFU focus. While this phantom study only considered one-dimensional (1D) motion, the authors plan to extend the system to 2D motion. In the system developed by An et al. [55], an optically tracked 2D ultrasound probe is handheld, and a *YK400XG* robot (YAMAHA) holds the HIFU transducer. The robot adapts the HIFU focus to the target position that is identified in the ultrasound images. In contrast to other systems, the treatment transducer, but not the ultrasound probe, is robot controlled. Another approach is proposed in [56] where a tracking accuracy study is performed. Here, two 2D ultrasound probes mounted on the HIFU transducer are used to track the target position using image registration with preinterventional image data. So far, the ultrasound probes and the transducer are static, but the authors plan to use a dual-robot system to reach higher flexibility in the future.

#### Radiation Therapy

In radiation therapy, tumors are treated by using ionizing radiation. Especially treatment of soft-tissue tumors is a challenging task due to organ motion [6]. For example, various approaches have been proposed to track tumor motion and adapt the radiation beam using ultrasound guidance [57, 58•]. However, in the treatment room, the probe needs to be placed on the patient for image acquisition. To help the operator with this task, Şen et al. [59] proposed an autonomous robotic ultrasound-guided patient alignment. Kuhlemann et al. [60] proposed a robotic camera-based patient localization approach where a depth camera is used to localize the patient within the treatment room and register the body surfaces from the preinterventional CT and the depth camera. In addition, optimal ultrasound view ports were calculated from the preinterventional CT. For treatment delivery, Schlüter et al. [61] proposed the usage of a kinematically redundant robot (*LBR iiwa*) to be able to avoid beam interferences caused by the robotic system and developed strategies for automatic ultrasound probe placement [62••]. In addition, safety aspects need to be considered [63] to prevent collisions and ensure that robot forces do not exceed acceptable values.

#### Summary

Autonomous therapy guidance systems are highly application-specific and depend on the ultrasound image analysis capability. While robotic motion compensation can already be performed using force sensitive robots, the automatic detection of target motion in 2D and 3D ultrasound images is still under active research. Furthermore, most evaluations were limited to phantom experiments, highlighting the need for more realistic in vivo studies.

## Trends and Future Directions

Trends in robotic ultrasound are focused on enhancing the autonomy of image acquisition, diagnosis, and therapy guidance. More advanced solutions are needed to supersede, for example, manually selected start and end points on/in the patient’s body. This could be achieved by using a body atlas including segmented organs based on MRI data. Furthermore, the capability to compensate for high-dimensional target motion and deformations should be improved to avoid target visibility loss in ultrasound images. The integration of ultrasound robots into the clinical workflow is also still under investigation. In this context, the interaction between the robot, operator, patient, and also safety aspects such as collision avoidance should be improved and have to be evaluated in in vivo studies. This could be achieved by using robots with at least six DOF and internal force sensors and additionally employing AI for robot navigation and image analysis. Another approach could be the use of VR and AR to create virtual environments and project the ultrasound image information directly into the field of view of the operator.

### Towards Intelligent Systems Using Artificial Intelligence

Even though there are several groups working towards autonomous systems (Table 2), the highest LORA observed in this review was seven. This might change during the next years due to the recent emerge of technologies in the field of AI.

From our point of view, there are two main application areas of AI to increase the autonomy of robotic ultrasound systems in the future: image understanding and robot navigation. For image understanding, CNNs showed exceptional performance in medical image analysis recently [64] and were successfully applied to ultrasound images [65]. An intelligent image understanding system can aim for enhanced navigation (e.g., in automatic landmark detection [66]), for diagnosis based on the acquired images (e.g., in the autonomous detection of a specific disease [67]), or for the identification of the individually optimal therapy [68]. Regarding autonomous robot navigation, deep reinforcement learning (DRL) [69] led to a breakthrough in robot learning such as human-aware path planning [70], object manipulation [71], and obstacle avoidance in complex dynamical environments [72]. Additionally, DRL provided promising results in its application for landmark detection in ultrasound images [73] and hence might also be interesting for image understanding. These approaches might play a key role in completely autonomously solving the ultrasound probe placement task, which remains one of the open challenges in autonomous robotic ultrasound system development.

### Virtual Reality and Augmented Reality

**Fig. 5:**
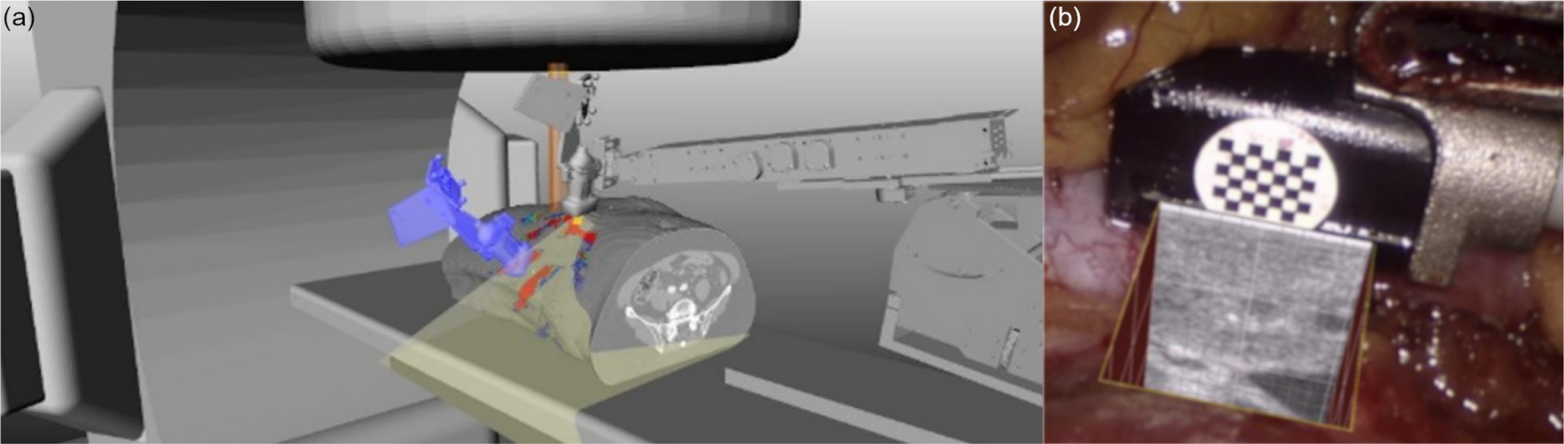
Examples of VR and AR in robotic ultrasound. **a** Virtual radiotherapy scenario showing a linear accelerator and the robotic ultrasound acquiring data from a patient (copyright [2016] John Wiley & Sons, Inc. Used with permission from [78] and John Wiley & Sons, Inc.). **b** 2D ultrasound image superimposed on a laparoscopic video image (reprinted from [79], copyright [2014] with permission from Elsevier)

**Fig. 6:**
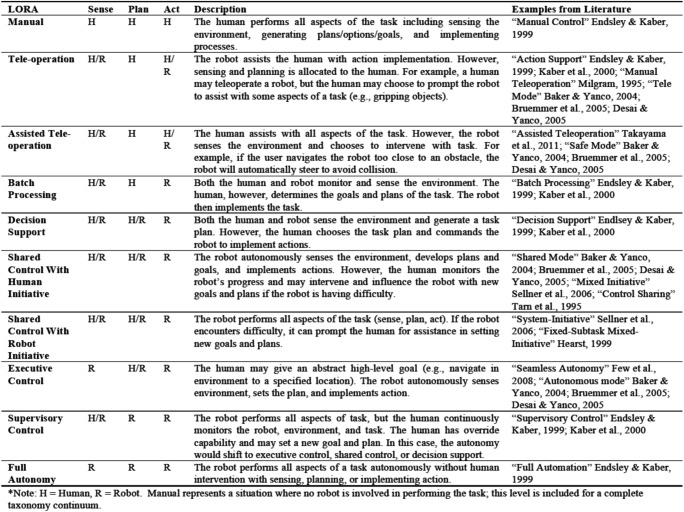
The applied taxonomy for levels of robot autonomy (figure by JM. Beer et al. [5] under CC-BY license)

In VR, a purely digital environment is generated with or without full user immersion, while AR refers to a real-world environment enhanced by means of overlying virtual content. Previous research reported the combination of these technologies and robotic ultrasound. Regarding VR, ultrasound data were displayed on graphical user interfaces for navigation [51•, 74, 75]. The virtual scenes were extended with 3D models of the robot that controlled the ultrasound probe for treatment guidance [76] and for simulation and/or verification of the robot setup (Fig. 5a) [77, 78]. The visualization of these virtual environments on head-mounted displays (HMDs) for a fully immersed experience seems logical to mimic a real experience. Regarding AR, the real scene was enhanced by means of 2D ultrasound images (Fig. 5b) [79], 3D ultrasound images [80], and tumor models from reconstructed ultrasound volumes [81–83]. The AR display technologies involved projection onto the organ surface [81], video see-through devices (specifically, remote consoles for surgical robots [82] and HMDs [83]), and optical see-through HMDs (specifically, *HoloLens* glasses [80]). These AR setups have a high potential to increase ergonomics since the sonographers can look at the patient while acquiring ultrasound images. The new developments in ultrasound probes, non-linear image registration, and VR/AR technologies (specifically, visualization techniques, sensor integration, and user interactions) open new opportunities in robotic ultrasound to enhance physician perception with subsurface targets and critical structures and also to improve 3D understanding.

## Conclusions

This review provides an overview of robotic ultrasound systems published within the last five years. Based on a standardized classification scheme for the autonomy level of a robotic system, each system was rated and categorized as a teleoperated, a collaborative assisting, or an autonomous system.

Teleoperated systems are developed sufficiently to perform remote exams at varying distances which is also supported by the fact that commercial systems are available nowadays. Current research on collaborative assisting systems focuses on ways to support the sonographer during the examination by means of probe positioning, navigation, and more intuitive visualizations. These systems may improve the quality of ultrasound acquisitions while providing more comfort and decreasing the mental load for the sonographer. As in other disciplines, autonomous systems are of special interest for robotic ultrasound systems as they could ultimately eliminate operator dependency. The review showed a wide variety of potential application fields, while research in these areas is still focused on ultrasound image processing as well as force adaptation strategies. In our opinion, a missing step is research on robust and reliable navigation and safety strategies for closed-loop applications to eventually reach full autonomy. Currently, the highest LORA of seven in this review shows that autonomous operation has not yet been achieved with robotic ultrasound. At the same time, many groups have declared a higher level of autonomy as their future project goal.

Future trends such as AI have the potential to increase autonomy of these platforms, with published work showing the promising capabilities of this technology in the fields of image understanding and robot navigation. At the same time, VR and AR technologies may improve ergonomics as well as spatial and anatomical understanding as these techniques allow displaying not only of important structures but also of the generated ultrasound image within the area of interest.

Overall, current robotic ultrasound systems show the potential to provide improved examination and intervention quality as well as a more ergonomically friendly work environment for sonographers with reduced workload. However, especially in this applied medical context, clinical studies are mandatory to assess the ultimate improvements in clinical outcomes.
